# Comorbidities of nontuberculous mycobacteria infection in Korean adults: results from the National Health Insurance Service–National Sample Cohort (NHIS–NSC) database

**DOI:** 10.1186/s12890-022-02075-y

**Published:** 2022-07-23

**Authors:** Seung Won Lee, Youngmok Park, Sol Kim, Eun Ki Chung, Young Ae Kang

**Affiliations:** 1 Institute of Immunology and Immunological Disease, Yonsei University College of Medicine, Seoul, Republic of Korea; 2 Division of Pulmonary and Critical Care Medicine, Department of Internal Medicine, Severance Hospital, Yonsei University College of Medicine, 50-1, Yonsei-ro, Seodaemun-gu, Seoul, Republic of Korea

**Keywords:** Nontuberculous mycobacteria infection, Comorbidities, Respiratory diseases

## Abstract

**Background:**

The global prevalence and incidence of nontuberculous mycobacteria (NTM) infection are increasing. However, the prevalence of NTM infection-associated comorbidities remains understudied. Thus, we investigated the comorbidities associated with NTM infection using the National Health Insurance Service-National Sample Cohort (NHIS–NSC) 2.0 database of the National Health Insurance Service (NHIS).

**Methods:**

In this case–control study, patients with NTM infection and controls aged 20–89 years of age were matched 1:4 by sex, age, region, and income. A total of 26 comorbidities were selected based on previous reports and claims data analysis. The distribution of comorbidities was compared between patients with NTM infection and controls by sex and age using logistic regression analysis.

**Results:**

In total, 893 patients (379 men and 514 women) with NTM infection (mean age, 56.1 years) and 3,572 controls (mean age, 55.6 years) were included. The odds ratio for prevalence of respiratory diseases, metabolic diseases, musculoskeletal disorders, gastrointestinal diseases, skin diseases, mental diseases, and neoplasms was significantly higher in patients with NTM infection than in the control group. Among comorbid diseases, the odds ratios (ORs) for the prevalence of the respiratory diseases such as bronchiectasis (OR [95% confidence interval (CI)]: 26.79 [19.69–36.45]) and interstitial pneumonitis (OR [95% CI]: 15.10 [7.15–31.89]) were the highest. No significant differences were observed in NTM infection-related comorbidities between men and women. In the younger age group (20–39 years old), the prevalence of respiratory and systemic diseases such as hypertension and diabetes was higher in the patient group than in the control group.

**Conclusions:**

NTM infection is associated with several respiratory and systemic diseases that should be considered when providing medical care to patients with NTM infection.

**Supplementary Information:**

The online version contains supplementary material available at 10.1186/s12890-022-02075-y.

## Background

Nontuberculous mycobacteria (NTM) infection is a primary cause of pulmonary disease and is recognized as a significant global public health issue due to its increasing prevalence in recent decades [1–3]. The growing prevalence of NTM infection has led to increased research on NTM infection-related comorbidities. A recent Japanese study reported that aspergillosis, asthma, chronic heart failure, diffuse pan-bronchiolitis, gastroesophageal reflux, interstitial pneumonia, cancers, and rheumatoid arthritis may be associated with NTM infection [4–6]. A previous study in the United States using health insurance claims data reported that patients with NTM infection had a higher prevalence of asthma, bronchiectasis, chronic obstructive pulmonary disease (COPD), arrhythmias, coronary artery disease, heart failure, and cancer compared to age- and sex-matched normal controls [1]. A German study also reported that COPD and emphysema were the most common diseases associated with NTM infection [7]. The prevalence of NTM infection-related diseases and comorbidities may differ by country, region, and race, given that NTM infection is caused by environmental pathogens prevalent in soil and water systems [2, 3, 8]. Further, regional characteristics or differences in lifestyles may lead to differences in the prevalence of NTM infection-related diseases and comorbidities.

Comorbidities are a risk factor for the development or progression of NTM infection [3]. Comorbidities can contribute to poor quality of life and more adverse events during multidrug antimycobacterial treatment [1, 9, 10]. NTM infection is a chronic condition, and patients with NTM infection tend to be older individuals with multiple comorbidities [6]. As such, a deeper understanding of comorbidities in patients with NTM infection is essential for clinicians to ensure appropriate initiation of NTM treatment and control of adverse drug reactions during treatment.

Despite reports of an increase in the prevalence and incidence of NTM infection in South Korea [2, 11–15], comorbidities associated with NTM infection remain understudied. Therefore, in this study, NTM infection-related comorbidities were investigated according to sex and age using national health insurance claims data in South Korea.

## Methods

### Data source

This study used data from the National Health Insurance Service-National Sample Cohort (NHIS–NSC) 2.0 database of the National Health Insurance Service (NHIS), a compulsory single-payer national health care coverage system in South Korea. The NHIS–NSC is a large-scale, population-based cohort comprising a representative sample of approximately 2% of the general Korean population. The database contains a de-identified research dataset including demographic information, disease diagnoses, therapeutic procedures, and drug prescriptions. In addition, the NHIS requires biennial health screening tests that include health questionnaire surveys, physical examinations, and laboratory tests. A detailed description of these data has been reported elsewhere [16].

### Study population

**Fig. 1:**
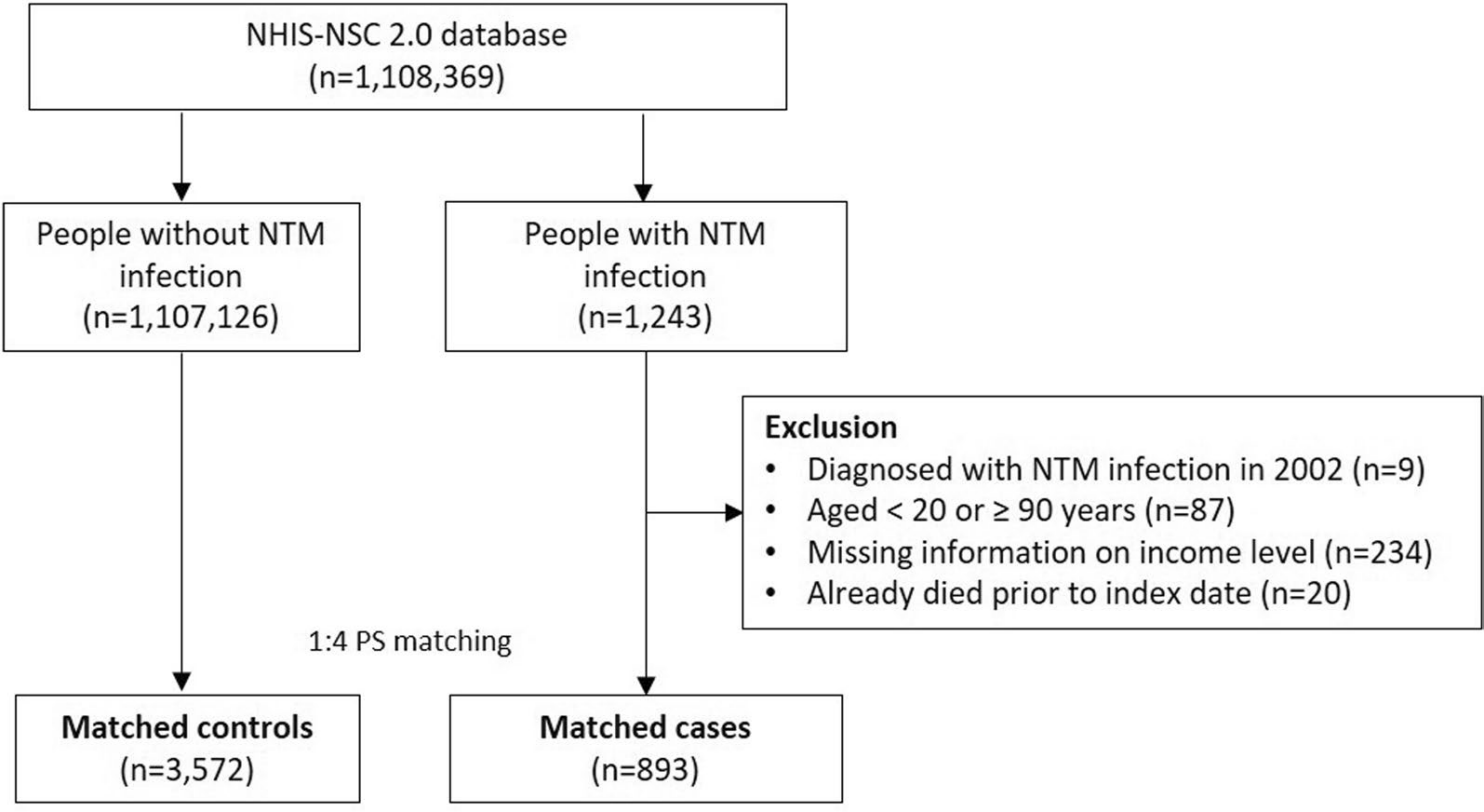
Flow diagram of the study population. (NTM, nontuberculous mycobacteria; PS, propensity score)

To investigate the comorbidities associated with NTM infection using the NHIS–NSC 2.0 database from 2002 to 2015, a case–control study was conducted. Figure 1 displays the selection process for the study population. Individuals with two or more claims diagnosed with NTM infection (International Classification of Disease Tenth Revision [ICD-10] A31) between January 2002 and December 2015 were identified from NHIS–NSC 2.0 data (*n* = 1,243). Among these individuals, we excluded nine patients with a history of NTM infection in 2002 to ensure inclusion only of patients with newly developed NTM infection. Additionally, we excluded individuals under the age of 20 or over 90 years (*n* = 87), without income information (*n* = 234), or who died before the index date (*n* = 20). The date of the initial claim for each patient's NTM infection was defined as the index date.

The selected patients with NTM were matched by sex, age, region of residence, and level of household income in the year of the index date with four controls who had no claims related to NTM infection from 2002 to 2015 using propensity score (PS) matching. PS matching reduced potential confounders and balanced the baseline covariates of the two groups [17]. PSs were derived from the predicted probability of patients with NTM infection versus those without NTM infection using a logistic regression model with adjustment for confounding by age, sex, region of residence, and household income. To reduce immortal time bias, a control group was set according to the characteristics corresponding to the year of diagnosis of patients with NTM infection [18]. A final total of 893 patients with NTM and 3,572 matched controls were included in the study. This study was approved by the Institutional Review Board of Severance Hospital (4–2020-1473) and Korea NHIS Medical Information Disclosure Committee (NHIS–2021–2-227). All methods were performed in accordance with the approved guidelines and regulations. Informed consent was waived, as this is a retrospective study of deidentified administrative data.

### Comorbidities and matching variables

In this study, diseases that were previously frequently reported as comorbidities in patients with NTM infection or were known risk factors were included as comorbidities of interest [1, 4, 6, 19, 20]. We also included a list of the most frequently claimed disease codes by examining the frequency of all disease codes claimed by patients with NTM infection from NHIS–NSC 2.0 data. Consequently, we investigated the distribution of 26 comorbidities potentially associated with NTM infection (Table 1). Comorbidities were identified with the ICD-10 code when there were two or more claims, and only claims up to 1 year before the index date were included. All methods were performed in accordance with the approved guidelines and regulations. Informed consent was waived, as this is a retrospective study of deidentified administrative data.

**Table 1:** ICD-10 codes for definition of comorbidities for nontuberculous mycobacterial infection

Comorbidities	ICD-10 codes
Diseases of the circulatory system	
Hypertension	I10.x, I11.x, I12.x, I15.x
Chronic heart failure	I11.x, I42.x, I50.x
Ischemic heart disease	I20.x, I21.x, I24.x, I25.1, I25.2, I25.5, I25.6, I25.9
Arrhythmia	I44.x, I45.x, I47.x, I48.x, I49
Endocrine, nutritional, and metabolic diseases	
Diabetes mellitus	E10.x, E11.x, E12.x, E13.x, E14.x, R73.0
Dyslipidemia	E78.x
Diseases of the respiratory system	
Acute sinusitis	J01, J01.x
Chronic sinusitis	J32, J32.x
Chronic obstructive pulmonary disease	J43.x, J44.9
Diffuse pan-bronchiolitis	J44.8
Asthma	J45.x
Bronchiectasis	J47
Interstitial pneumonia	J84.1, J84.9, J70.4
Diseases of the musculoskeletal system	
Rheumatoid arthritis	M06.9
Osteoporosis	M80.x, M81.x
Bone fracture	M80.x, M84.0, M84.3, M84.4, M96.6, S02.x, S22.x, S32.x, S42.x, S52.x, S62.x, S72.x, S82.x, S92.x, T02.x, T08, T14.2, T91.1, T93.2, T94.1
Diseases of the digestive system	
Chronic viral hepatitis	B18.1, B18.2, K70.3, K74.6
Gastroesophageal reflux disease	K21.0, K21.9
Diseases of the genitourinary system	
Chronic kidney disease	N18.x, N28.9
Diseases of the skin and subcutaneous tissue	
Atopic dermatitis	L20, L20.8, L20.9
Seborrheic dermatitis	L21
Contact dermatitis	L23, L23.x, L24, L24.x, L25
Other dermatitis	L30, L30.x
Urticaria	L50, L50.x, L50.8x
Mental and behavioral disorders	Included all F codes
Neoplasms	Included all C codes

To identify diseases frequently associated with age, age was classified into four groups (20–39, 40–59, 60–79, and 80–89 years old). The income groups initially comprising 11 classes (class 0, lowest income; class 10, highest income) in the NHIS database were recategorized into three groups (low, class 0–2; medium, class 3–7; high, class 8–10). Region of residence was recategorized into urban (Seoul, Busan, Daegu, Incheon, Gwangju, Daejeon, and Ulsan) and rural (Gyeonggi, Gangwon, Chungcheongbuk, Chungcheongnam, Jeollabuk, Jeollanam, Gyeongsangbuk, Gyeongsangnam, and Jeju).

### Statistical analysis

General characteristics were compared between patients with NTM infection and the control group using an independent *t*-test for continuous variables and the chi-squared test for categorical variables. To compare the distribution of comorbidities between control and NTM groups, logistic regression was used to calculate odds ratio (OR) and 95% confidence intervals (CI). Subgroup analyses according to sex and age were also conducted. All analyses were conducted using SAS Enterprise Guide 7.1 (SAS Institute Inc., Cary, NC, USA). Statistical significance was defined as a two-sided *P* value of < 0.05.

## Results

### Characteristics of the study population

**Fig. 2:**
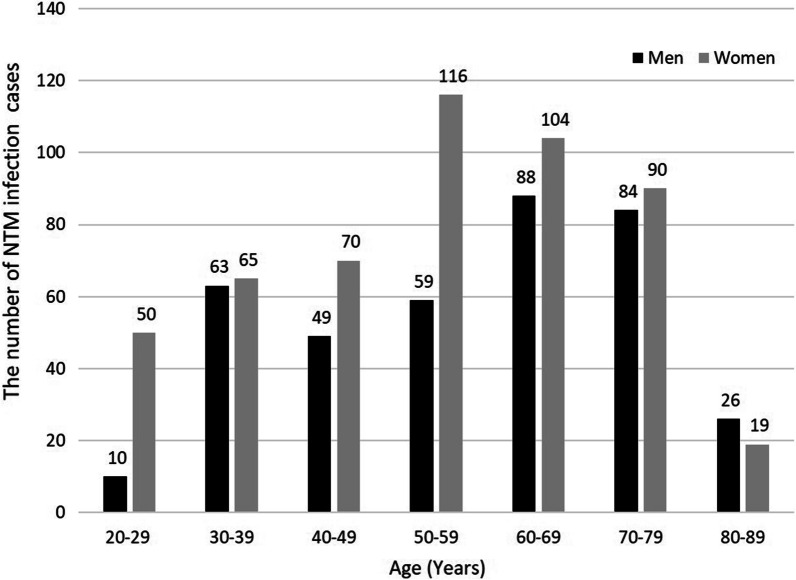
The distribution of the patients with NTM infection by sex and age. (NTM, nontuberculous mycobacteria)

The general characteristics of the study population are presented as a whole and according to sex in Table 2. In total, 893 patients with NTM infection (mean age, 56.1 years) and 3572 controls (mean age, 55.6 years) were included. Among patients with NTM infection, 379 (42.4%) were men and 514 (57.6%) were women, with mean ages of 57.9 and 54.3 years, respectively. Classification according to sex and age revealed a higher proportion of female patients than male patients, except for patients in their 80 s. The most common age was 50–59 years (22.6%) for women and 60–69 years (23.2%) for men (Fig. 2). No significant differences were observed in sex, age, income, or region between patients with NTM infection and controls after PS matching. However, a significant difference was observed in the number of comorbidities between patients with NTM infection and controls. Comparison of the number of comorbidities between the NTM and control groups revealed that 54% of the control group had fewer than five comorbidities, whereas 68.4% of the NTM group had five or more comorbidities. Similar results were obtained in the analysis according to sex.

**Table 2:** Baseline characteristics of the study population after propensity score matching

	Total			Men			Women		
	NTM (*N* = 893)^a^	Non-NTM (*n* = 3572)^a^	*p* value	NTM (*n* = 379)^a^	Non-NTM (*n* = 1516)^a^	*p* value	NTM (*n* = 514)^a^	Non-NTM (n = 2056)^a^	*p* value
Sex			1.0000			NA			NA
Male	379 (42.4)	1516 (42.4)		NA	NA		NA	NA	
Female	514 (57.6)	2056 (57.6)		NA	NA		NA	NA	
Age, years	56.1 ± 16.7	55.6 ± 16.5	0.7256	57.9 ± 16.2	57.6 ± 16.2	0.6949	54.3 ± 16.4	54.2 ± 16.7	0.8951
Age group			1.0000			1.0000			1.0000
20–29	60 (6.7)	240 (6.7)		10 (2.6)	40 (2.6)		50 (9.7)	200 (9.7)	
30–39	128 (14.3)	512 (14.3)		63 (16.6)	252 (16.6)		65 (12.6)	260 (12.6)	
40–49	119 (13.3)	476 (13.3)		49 (12.9)	196 (12.9)		70 (13.6)	280 (13.6)	
50–59	175 (19.6)	700 (19.6)		59 (15.6)	236 (15.6)		116 (22.6)	464 (22.6)	
60–69	192 (21.5)	768 (21.5)		88 (23.2)	352 (23.2)		104 (20.2)	416 (20.2)	
70–79	174 (19.5)	696 (19.5)		84 (22.2)	336 (22.2)		90 (17.5)	360 (17.5)	
80–89	45 (5.0)	180 (5.0)		26 (6.9)	104 (6.9)		19 (3.7)	76 (3.7)	
Income^b^			1.0000			1.0000			1.0000
Low (0–2)	- (0.0)	- (0.0)		- (0.0)	- (0.0)		- (0.0)	- (0.0)	
Medium (3–7)	394 (44.1)	1576 (44.1)		174 (45.9)	696 (45.9)		220 (42.8)	880 (42.8)	
High (8–10)	499 (55.9)	1996 (55.9)		205 (54.1)	820 (54.1)		294 (57.2)	1176 (57.2)	
Region of residence^c^			1.0000			1.0000			1.0000
Urban	414 (46.4)	1656 (46.4)		150 (39.6)	600 (39.6)		264 (51.4)	1056 (51.4)	
Rural	479 (53.6)	1916 (53.6)		229 (60.4)	916 (60.4)		250 (48.6)	1000 (48.6)	
No. of comorbidities			< 0.0001			< 0.0001			< 0.0001
0	28 (3.1)	349 (9.8)		21 (5.5)	200 (13.2)		7 (1.4)	149 (7.2)	
1–4	240 (26.9)	1579 (44.2)		105 (27.7)	694 (45.8)		135 (26.3)	885 (43.0)	
5–9	400 (44.8)	1224 (34.3)		166 (43.8)	449 (29.6)		234 (45.5)	775 (37.7)	
≥ 10	225 (25.2)	420 (11.8)		87 (23.0)	173 (11.4)		138 (26.8)	247 (12.0)	

^a^Data are presented as mean ± standard deviation or number (%)

^b^Income was divided into 11 classes (class 1, lowest income; class 11, highest income), which were reclassified into three groups (low, class 0–2; medium, class 3–7; high, 8–10). After propensity score matching, no individuals were in the “low (class 0–2)” group

^c^Region of residence was categorized into urban (Seoul, Busan, Daegu, Incheon, Gwangju, Daejeon, and Ulsan) and rural (Gyeonggi, Gangwon, Chungcheongbuk, Chungcheongnam, Jeollabuk, Jeollanam, Gyeongsangbuk, Gyeongsangnam, and Jeju)

*NTM,* Nontuberculous mycobacteria infection

### Prevalence of comorbidities in patients with NTM infection

**Fig. 3:**
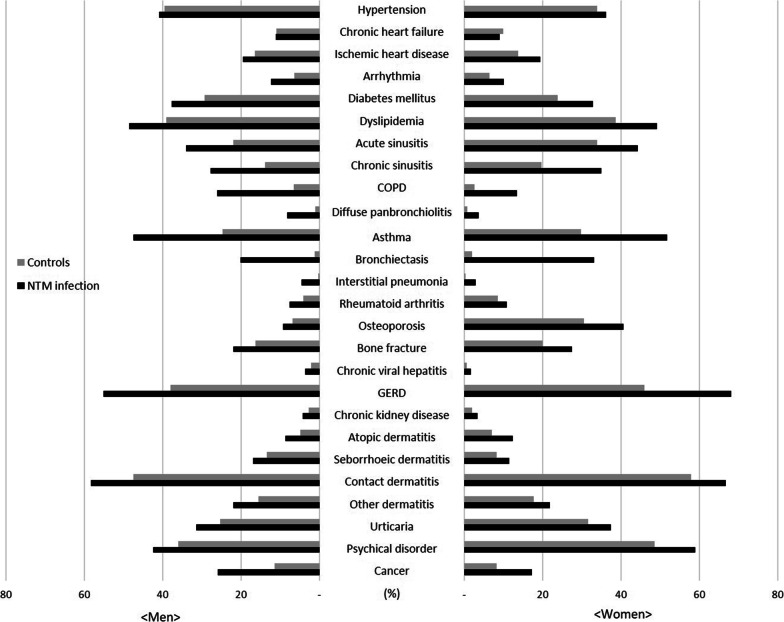
Frequency (%) of NTM infection-associated comorbidities. (COPD, chronic obstructive pulmonary disease; GERD, gastroesophageal reflux disease; NTM, non-tuberculous mycobacteria)

With regard to the prevalence of comorbidities in the entire patient group, the frequency of contact dermatitis (63.2%) was the highest, followed by gastroesophageal reflux disease (GERD, 62.6%) and mental and behavioral disorders (52.0%). Similar results were observed in the control group (Table 3). In men, contact dermatitis (58.3%), GERD (55.1%), hyperlipidemia (48.5%), asthma (47.5%), and psychiatric disorders (42.5%) were the most common, whereas in women, GERD (68.1%) was the most common, followed by contact dermatitis (58.3%), mental disorders (58.9%), asthma (51.8%), and hyperlipidemia (49.2%) (Table 4 and Fig. 3).

**Table 3:** Comorbidities of interest for nontuberculous mycobacteria infection in total study population matching for age, sex, income, and region

Comorbidities	NTM (*N* = 893) *n*(%)		Non-NTM (*N* = 3572) *n*(%)		Odds ratio [95% CI]	*p* value
Diseases of the circulatory system						
Hypertension	341 (38.2)		1297 (37.3)		1.10 [0.92–1.32]	0.2922
Chronic heart failure	88 (9.9)		371 (10.9)		0.93 [0.71–1.20]	0.5685
Ischemic heart disease	174 (19.5)		533 (15.4)		1.45 [1.18–1.78]	0.0005
Arrhythmia	99 (11.1)		228 (6.6)		1.87 [1.45–2.41]	< 0.0001
Endocrine, nutritional, and metabolic diseases						
Diabetes mellitus	312 (34.9)		936 (26.9)		1.65 [1.38–1.96]	< 0.0001
Dyslipidemia	437 (48.9)		1388 (39.1)		1.62 [1.38–1.90]	< 0.0001
Diseases of the respiratory system						
Acute sinusitis	357 (40.0)		1029 (28.8)		1.67 [1.43–1.95]	< 0.0001
Chronic sinusitis	285 (31.9)		615 (17.2)		2.27 [1.92–2.68]	< 0.0001
COPD	168 (18.8)		155 (4.4)		6.42 [4.97–8.29]	< 0.0001
Diffuse pan-bronchiolitis	50 (5.6)		33 (0.9)		6.62 [4.21–10.42]	< 0.0001
Asthma	446 (49.9)		986 (27.9)		2.75 [2.36–3.22]	< 0.0001
Bronchiectasis	246 (27.5)		59 (1.8)		26.79 [19.69–36.45]	< 0.0001
Interstitial pneumonia	32 (3.6)		9 (0.2)		15.10 [7.15–31.89]	< 0.0001
Diseases of the musculoskeletal system						
Rheumatoid arthritis	85 (9.5)		238 (6.7)		1.49 [1.14–1.95]	0.0031
Osteoporosis	244 (27.3)		732 (20.9)		1.80 [1.45–2.23]	< 0.0001
Bone fracture	224 (25.1)		656 (18.8)		1.52 [1.27–1.81]	< 0.0001
Diseases of the digestive system						
Chronic viral hepatitis	23 (2.6)		45 (1.3)		2.07 [1.24–3.46]	0.0052
GERD	559 (62.6)		1521 (42.7)		2.35 [2.01–2.74]	< 0.0001
Diseases of the genitourinary system						
Chronic kidney disease	33 (3.7)		81 (2.4)		1.65 [1.09–2.51]	0.0176
Diseases of the skin and subcutaneous tissue						
Atopic dermatitis	97 (10.9)		220 (6.2)		1.86 [1.45–2.39]	< 0.0001
Seborrheic dermatitis	123 (13.8)		372 (10.5)		1.38 [1.10–1.71]	0.0045
Contact dermatitis	564 (63.2)		1909 (53.6)		1.50 [1.29–1.75]	< 0.0001
Other dermatitis	195 (21.8)		599 (16.8)		1.39 [1.16–1.66]	0.0004
Urticaria	311 (34.8)		1032 (28.9)		1.32 [1.13–1.54]	0.0006
Mental and behavioral disorders	464 (52.0)		1543 (43.8)		1.50 [1.28–1.76]	< 0.0001
Neoplasms	187 (20.9)		345 (10.1)		2.65 [2.16–3.26]	< 0.0001

*COPD,* Chronic obstructive pulmonary disease, *GERD,* Gastroesophageal reflux disease, *NTM,* Non-tuberculous mycobacteria infection. In the logistic regression model, sex, age, region of residence, and income level were adjusted

**Table 4:** Comorbidities of interest for nontuberculous mycobacteria infection according to sex matching for age, sex, income, and region

Comorbidities	Men				Women			
	NTM (*N* = 379)*	Non-NTM (*n* = 1516)*	Odds ratio [95% CI]	*p* value	NTM (*n* = 514)*	Non-NTM (*n* = 2056)*	Odds ratio [95% CI]	*p* value
Diseases of the circulatory system								
Hypertension	155 (40.9)	600 (39.6)	1.04 [0.80–1.37]	0.7587	186 (36.2)	697 (33.9)	1.16 [0.91–1.48]	0.2457
Chronic heart failure	42 (11.1)	167 (11.0)	0.98 [0.67–1.43]	0.9236	46 (8.9)	204 (9.9)	0.88 [0.62–1.27]	0.4977
Ischemic heart disease	74 (19.5)	249 (16.4)	1.24 [0.91–1.70]	0.1678	100 (19.5)	284 (13.8)	1.63 [1.24–2.15]	0.0005
Arrhythmia	47 (12.4)	96 (6.3)	2.17 [1.48–3.19]	< 0.0001	52 (10.1)	132 (6.4)	1.67 [1.18–2.35]	0.0035
Endocrine, nutritional, and metabolic diseases								
Diabetes mellitus	143 (37.7)	445 (29.4)	1.54 [1.19–2.01]	0.0011	169 (32.9)	491 (23.9)	1.74 [1.37–2.20]	< 0.0001
Dyslipidemia	184 (48.5)	592 (39.1)	1.55 [1.21–1.99]	0.0005	253 (49.2)	796 (38.7)	1.67 [1.35–2.07]	< 0.0001
Diseases of the respiratory system								
Acute sinusitis	129 (34.0)	332 (21.9)	1.84 [1.44–2.36]	< 0.0001	228 (44.4)	697 (33.9)	1.57 [1.29–1.92]	< 0.0001
Chronic sinusitis	105 (27.7)	211 (13.9)	2.38 [1.82–3.12]	< 0.0001	180 (35.0)	404 (19.6)	2.21 [1.79–2.73]	< 0.0001
COPD	99 (26.1)	100 (6.6)	6.66 [4.70–9.43]	< 0.0001	69 (13.4)	55 (2.7)	6.35 [4.32–9.34]	< 0.0001
Diffuse pan-bronchiolitis	31 (8.2)	16 (1.1)	8.77 [4.69–16.41]	< 0.0001	19 (3.7)	17 (0.8)	4.74 [2.43–9.24]	< 0.0001
Asthma	180 (47.5)	375 (24.7)	2.95 [2.31–3.76]	< 0.0001	266 (51.8)	611 (29.7)	2.64 [2.15–3.23]	< 0.0001
Bronchiectasis	76 (20.1)	19 (1.3)	20.97 [12.41–35.44]	< 0.0001	170 (33.1)	40 (1.9)	30.55 [20.88–44.71]	< 0.0001
Interstitial pneumonia	17 (4.5)	4 (0.3)	18.41 [6.09–55.65]	< 0.0001	15 (2.9)	5 (0.2)	12.58 [4.53–34.91]	< 0.0001
Diseases of the musculoskeletal system								
Rheumatoid arthritis	29 (7.7)	61 (4.0)	1.99 [1.25–3.18]	0.004	56 (10.9)	177 (8.6)	1.31 [0.95–1.81]	0.1031
Osteoporosis	35 (9.2)	104 (6.9)	1.40 [0.91–2.16]	0.1266	209 (40.7)	628 (30.5)	1.97 [1.53–2.52]	< 0.0001
Bone fracture	83 (21.9)	246 (16.2)	1.45 [1.09–1.92]	0.0102	141 (27.4)	410 (19.9)	1.59 [1.26–2.02]	0.0001
Diseases of the digestive system								
Chronic viral hepatitis	14 (3.7)	31 (2.0)	1.82 [0.96–3.48]	0.0688	9 (1.8)	14 (0.7)	2.60 [1.12–6.06]	0.0266
GERD	209 (55.1)	575 (37.9)	2.09 [1.65–2.65]	< 0.0001	350 (68.1)	946 (46.0)	2.57 [2.09–3.17]	< 0.0001
Diseases of the genitourinary system								
Chronic kidney disease	16 (4.2)	41 (2.7)	1.57 [0.86–2.86]	0.1402	17 (3.3)	40 (1.9)	1.73 [0.97–3.09]	0.0631
Diseases of the skin and subcutaneous tissue								
Atopic dermatitis	33 (8.7)	74 (4.9)	1.86 [1.21–2.85]	0.0046	64 (12.5)	146 (7.1)	1.86 [1.36–2.54]	< 0.0001
Seborrheic dermatitis	64 (16.9)	202 (13.3)	1.32 [0.97–1.80]	0.0816	59 (11.5)	170 (8.3)	1.44 [1.05–1.97]	0.0229
Contact dermatitis	221 (58.3)	720 (47.5)	1.57 [1.24–1.98]	0.0002	343 (66.7)	1189 (57.8)	1.46 [1.19–1.79]	0.0002
Other dermatitis	83 (21.9)	236 (15.6)	1.52 [1.15–2.03]	0.0037	112 (21.8)	363 (17.7)	1.30 [1.02–1.65]	0.0313
Urticaria	119 (31.4)	383 (25.3)	1.36 [1.06–1.74]	0.0169	192 (37.4)	649 (31.6)	1.29 [1.06–1.59]	0.0124
Mental and behavioral disorders	161 (42.5)	545 (35.9)	1.35 [1.05–1.74]	0.0175	303 (58.9)	998 (48.5)	1.61 [1.30–1.98]	< 0.0001
Neoplasms	98 (25.9)	174 (11.5)	3.07 [2.26–4.17]	< 0.0001	89 (17.3)	171 (8.3)	2.39 [1.80–3.17]	< 0.0001

^*^Data are presented as number (%). In the logistic regression model, sex, age, region of residence, and income level were adjusted

*COPD,* Chronic obstructive pulmonary disease, *GERD,* Gastroesophageal reflux disease, *NTM,* Nontuberculous mycobacteria infection

## Comparison of risk of comorbidities between NTM and control groups

### Unstratified analysis

Table 3 presents the results of logistic regression analysis of the association between NTM infection and comorbidities in the total population, adjusted for sex, age, income, and region. The prevalence of most comorbidities was significantly higher in patients with NTM infection than in the control group, except for hypertension and chronic heart failure. Among the respiratory diseases, the ORs for the prevalence of bronchiectasis (OR [95% CI]: 26.79 [19.69–36.45]), interstitial pneumonitis (OR [95% CI]: 15.10 [7.15–31.89]), diffuse pan-bronchiolitis (OR [95% CI]: 6.62 [4.21–10.42]), and COPD (OR [95% CI]: 6.42 [4.97–8.29]) were higher in patients with NTM infection than in controls.

### Sex-stratified analysis

Sex-stratified logistic regression analysis revealed that endocrine, nutritional, metabolic, and respiratory system diseases; mental and behavioral disorders; and neoplasms were significantly associated with NTM infection in both sexes (Table 4). However, circulatory and genitourinary diseases such as hypertension, chronic heart failure, and chronic kidney disease were not associated with NTM infection in both sexes. In both sexes, the OR for the prevalence of bronchiectasis was the highest (OR [95% CI]: 20.97 [12.43–35.44] in men and 30.55 [20.88–44.71] in women), followed by interstitial pneumonia (OR [95% CI]: 18.41 [6.09–55.65] in men and 12.58 [4.53–34.91] in women).

Ischemic heart disease (OR [95% CI]: 1.63 [1.24–2.15]), osteoporosis (OR [95% CI]: 1.97 [1.53–2.52]), chronic viral hepatitis (OR [95% CI]: 2.60 [1.12–6.06]), and seborrheic dermatitis (OR [95% CI]: 1.44 [1.05–1.97]) were associated with NTM infection only in women. In contrast, rheumatoid arthritis (OR [95% CI]: 1.99 [1.25–3.18]) was associated with NTM infection only in men.

### Age-stratified analysis

Analysis of the risk of comorbidities according to age group revealed that dyslipidemia, asthma, bronchiectasis, interstitial pneumonia, GERD, and cancers were significantly associated with NTM infection in all age groups (Additional file 1: Tables S1–S4). Arrhythmia, diabetes mellitus, acute sinusitis, diffuse pan-bronchiolitis, osteoporosis, atopic dermatitis, contact dermatitis, and mental and behavioral disorders were associated with NTM infection in all age groups except the 80–89 years age group.

Hypertension (OR [95% CI]: 3.58 [1.67–7.64]) was associated with NTM infection only in the 20–39 years age group, whereas rheumatoid arthritis (OR [95% CI]: 2.18 [1.35–3.50]) and chronic kidney disease (OR [95% CI]: 3.31 [1.35–8.09]) were associated with NTM infection only in the 40–59 years age group. Chronic viral hepatitis was not significantly associated with NTM infection in any age group. COPD was associated with NTM infection in all age groups except for the 20–29 years age group. Ischemic heart disease was associated with NTM in the 40–59 and 80–89 years age groups. In contrast, fractures were associated with NTM infection in all age groups except the 50–59 years age group, while seborrheic dermatitis and urticaria were associated with NTM infection in the 20–39 and 40–59 years age groups.

## Discussion

This case–control study investigated comorbidities associated with NTM infection according to sex and age using national claims data in Korea. Consistent with previous studies, respiratory diseases (bronchiectasis, COPD, interstitial pneumonia, asthma, and pan-bronchiolitis) and non-respiratory conditions (GERD and malignancies) were founded to be associated with NTM infection in this study [1, 2, 6]. A cohort study matched by sex, age, and insurance benefit coverage in the United States (U.S.) also reported that several respiratory diseases (such as asthma, bronchiectasis, and COPD) and systemic diseases such as mental disorders, metabolic diseases (diabetes mellitus and hyperlipidemia), musculoskeletal disorders, cardiovascular diseases (such as coronary artery diseases, myocardial infarction, and arrhythmia) and neoplasm were associated with NTM infection, similar to our findings [21]. In the unstratified analysis in our study, we identified significant associations between diffuse pan-bronchiolitis, interstitial pneumonia, osteoporosis, and chronic kidney disease and NTM infection, consistent with the results of a cross-sectional study in Japan [6].

Previous studies have reported that structural lung disease including bronchiectasis, COPD, and interstitial lung disease are risk factors for NTM infection [3, 22]. In structural lung disease, local damage in the lungs can result in non-clearing infections, leading to an excessive inflammatory response with further lung damage, a decline in lung function, and more severe infection [23]. In addition, patients with asthma and COPD treated with inhaled corticosteroids and patients with interstitial lung disease treated with systemic immunosuppressants were more likely to have NTM infection than those not treated with corticosteroids and systemic immunosuppressants [1–3, 24, 25]. These studies suggest that the use of corticosteroids and systemic immunosuppressants is associated with NTM infection.

No significant differences were observed in comorbidities between men and women in our study. Traditionally, the phenotype of NTM infection is considered to differ between men and women. The typical phenotype of right middle lobe and lingular segment involvement in NTM pulmonary disease is termed ‘Lady Windermere syndrome’ and is considered a phenotype that occurs frequently in post-menopausal, slender women [26, 27]. In contrast, the fibro-cavitary form of NTM pulmonary disease is considered a phenotype in men with COPD [28]. However, previous reports suggest a similar NTM phenotype between men and women [29, 30]. Although we could not confirm the phenotype in our study, we did not identify a significant difference in the distribution of comorbidities related to NTM infection between men and women.

Compared to a previous Japanese study [6], our study revealed several distinct findings regarding comorbidities in the sex-stratified analysis. Ischemic heart disease was associated with NTM infection in women in our study but not in the previous study in Japan. Conversely, rheumatoid arthritis was not associated with NTM infection in women in our study but was associated with NTM infection in the Japanese study. The study conducted in Japan only included individual aged < 75 years and dataset used was not the national representative dataset, but they had stricter definition of NTM than our study as those who were claimed 3 or more times as an A31 disease code, and the number of NTM patients as subjects was relatively small (men = 134, women = 285) compared to our study. The discrepancies in results between these studies could be partly underpinned by differences in race, lifestyle, study design, sample size, characteristics of claims data, and the prevalence of each comorbidity.

Among the 20–39 years age group, the prevalence of systemic diseases such as hypertension, diabetes mellitus, and malignancy, as well as respiratory diseases, which are traditional risk factors for NTM infection, was higher than that in the control group. Hypertension was associated with NTM infection in the U.S. study but not in the Japanese study [6, 31]. In our study, hypertension and NTM infection were associated with NTM infection only in the 20–39 years age group. Although we did not conclusively determine the reason for the association between hypertension and NTM infection only in younger age groups, a possibility is that NTM infection is associated with diastolic blood pressure (DBP) rather than systolic blood pressure (SBP). Indeed, DBP increases in most hypertensive patients before 50 years of age, after which SBP continues to rise and DBP tends to decrease due to a decrease in elasticity of blood vessel walls and blood volume with aging. This condition is referred to as isolated systolic hypertension [32, 33]. A relationship between hypertension and NTM infection may not be observed in individuals over the age of 50 years, the timepoint at which isolated systolic hypertension begins to increase [32, 33].

Consistent with our findings, depression was associated with NTM infection in the U.S. study, but this association was not observed in the Japanese study [1, 6]. Anxiety, depression, and poor sleep quality are frequently observed in patients with NTM infection [34–36]. Our study suggests that mental disorders such as depression may increase the risk of NTM infection.

Several limitations of the present study should be acknowledged. First, the diagnosis of NTM infection and comorbidities in this study was based on insurance claims, which rely on ICD-10 codes. The use of administrative claims data may have resulted in issues such as coding accuracy (over-coding, under-coding, or miscoding), diagnostic inertia, and lack of disease specificity. Thus, we defined the disease based on at least two claims, similar to most previous studies analyzing claims data [1, 2, 31, 37, 38]. Second, it was impossible to classify and analyze cases of infectious diseases such as tuberculosis, mental/behavioral disorders, or cancer in the NHIS–NSC 2.0 database because the detailed disease code was partially disclosed to protect personal information. In addition, human immunodeficiency virus and cystic fibrosis are known risk factors for NTM infection [23], but these diseases are classified as personal sensitive information in the NHIS–NSC 2.0 database and are therefore not disclosed. As such, we were unable to confirm their relevance to NTM infection. Third, the mycobacterium species could not be distinguished because of the unavailability of microbial information. Fourth, possible residual confounding factors, including dietary, personal lifestyle, clinical, or other environmental factors may affect the association between NTM infection and comorbidities. Finally, the study population was homogeneously Korean, which may limit the generalizability of the findings to other ethnic populations.

Despite these limitations, our study has several strengths. First, this study used the NHIS–NSC 2.0 database of South Korea, which is representative of the entire population and contains healthcare utilization information from all settings in South Korea. Second, to our knowledge, this is the first study in Korea to examine the comorbidities associated with NTM infection according to sex and age. Region of residence and income level were also considered to be factors that would affect the relationship between NTM infection and comorbidities; hence, matching and logistic regression analysis were conducted taking this into account. Third, we investigated the ranking of comorbidities with high frequency in patients with NTM infection using preliminary analysis of claims data and added chronic sinusitis, acute sinusitis, and skin diseases to the comorbidities investigated in previous studies.

## Conclusions

In conclusion, our data revealed NTM infection-associated comorbidities in Korean adults. Our data suggests the possibility that providing adequate medical care to patients with these comorbidities may be important in preventing NTM infection. Clinicians should consider common multiple comorbidities in the management of patients with NTM infection.

## Supplementary Information

**Additional file 1: Table S1.** Comorbidities of nontuberculous mycobacteria infection according to age group (20-39 years old). **Table S2.** Comorbidities of nontuberculous mycobacteria infection according to age group (40-59 years old). **Table S3. **Comorbidities of nontuberculous mycobacteria infection according to age group (60-79 years old). **Table S4.** Comorbidities of nontuberculous mycobacterial infection according to age group (80-89 years old)

## Data Availability

Release of the data by the researchers is not legally permitted. All of the data are available from the database of National Health Insurance Sharing Service (NHISS) (available from: https://nhiss.nhis.or.kr/bd/ay/bdaya001iv.do). NHISS allows the data to be used by any researcher who agrees to abide by the research ethics with some cost. The data for this article can be accessed and downloaded from the website after agreeing to abide by the research ethics.

## Abbreviations

CI: Confidence interval
COPD: Chronic obstructive pulmonary disease
DBP: Diastolic blood pressure
GERD: Gastroesophageal reflux disease
ICD: International classification of disease tenth revision
NHIS–NSC: National health insurance service-national sample cohort
NTM: Nontuberculous mycobacteria
OR: Odds ratio
PS: Propensity score
SBP: Systolic blood pressure

## References

[1] Marras TK, Vinnard C, Zhang Q, Hamilton K, Adjemian J, Eagle G, Zhang R, Chou E, Olivier KN (2018). Relative risk of all-cause mortality in patients with nontuberculous mycobacterial lung disease in a US managed care population. Respir Med.

[2] Kim HO, Lee K, Choi HK, Ha S, Lee SM, Seo GH (2019). Incidence, comorbidities, and treatment patterns of nontuberculous mycobacterial infection in South Korea. Medicine.

[3] Prevots DR, Marras TK (2015). Epidemiology of human pulmonary infection with nontuberculous mycobacteria: a review. Clin Chest Med.

[4] Tsuji T, Tanaka E, Yasuda I, Nakatsuka Y, Kaji Y, Yasuda T, Hashimoto S, Hwang MH, Hajiro T, Taguchi Y (2015). Nontuberculous mycobacteria in diffuse panbronchiolitis. Respirology.

[5] Kusumoto T, Asakura T, Suzuki S, Okamori S, Namkoong H, Fujiwara H, Yagi K, Kamata H, Ishii M, Betsuyaku T (2019). Development of lung cancer in patients with nontuberculous mycobacterial lung disease. Respir Investig.

[6] Uno S, Asakura T, Morimoto K, Yoshimura K, Uwamino Y, Nishimura T, Hoshino Y, Hasegawa N (2020). Comorbidities associated with nontuberculous mycobacterial disease in Japanese adults: a claims-data analysis. BMC Pulm Med.

[7] Ringshausen FC, Wagner D, de Roux A, Diel R, Hohmann D, Hickstein L, Welte T, Rademacher J (2016). Prevalence of nontuberculous mycobacterial pulmonary disease, Germany, 2009–2014. Emerg Infect Dis.

[8] Sekikawa A, Ueshima H, Kadowaki T, El-Saed A, Okamura T, Takamiya T, Kashiwagi A, Edmundowicz D, Murata K, Sutton-Tyrrell K (2007). Less subclinical atherosclerosis in Japanese men in Japan than in white men in the United States in the post–World War II birth cohort. Am J Epidemiol.

[9] Asakura T, Funatsu Y, Ishii M, Namkoong H, Yagi K, Suzuki S, Asami T, Kamo T, Fujiwara H, Uwamino Y (2015). Health-related quality of life is inversely correlated with C-reactive protein and age in Mycobacterium avium complex lung disease: a cross-sectional analysis of 235 patients. Respir Res.

[10] Diel R, Lipman M, Hoefsloot W (2018). High mortality in patients with Mycobacterium avium complex lung disease: a systematic review. BMC Infect Dis.

[11] Yoon HJ, Choi HY, Ki M (2017). Nontuberculosis mycobacterial infections at a specialized tuberculosis treatment centre in the Republic of Korea. BMC Infect Dis.

[12] Kim JK, Rheem I (2013). Identification and distribution of nontuberculous mycobacteria from 2005 to 2011 in cheonan. Korea Tubercul Respir Diseases.

[13] Kwon YS, Koh W-J (2014). Diagnosis of pulmonary tuberculosis and nontuberculous mycobacterial lung disease in Korea. Tubercul Respir Diseases.

[14] Koh W-J, Chang B, Jeong B-H, Jeon K, Kim S-Y, Lee NY, Ki C-S, Kwon OJ (2013). Increasing recovery of nontuberculous mycobacteria from respiratory specimens over a 10-year period in a tertiary referral hospital in South Korea. Tubercul Respir Diseases.

[15] Ko R-E, Moon SM, Ahn S, Jhun BW, Jeon K, Kwon OJ, Huh HJ, Ki C-S, Lee NY, Koh W-J: Changing epidemiology of nontuberculous mycobacterial lung diseases in a tertiary referral hospital in Korea between 2001 and 2015. J Korean Med Sci 2018, 10.3346/jkms.2018.33.e65. 10.3346/jkms.2018.33.e65 PMC5809754 29441757

[16] Lee J, Lee JS, Park SH, Shin SA, Kim K (2017). Cohort profile: the national health insurance service-national sample cohort (NHIS–NSC), South Korea. Int J Epidemiol.

[17] Austin PC (2011). An Introduction to propensity score methods for reducing the effects of confounding in observational studies. Multivariate Behav Res.

[18] Woo A, Lee SW, Koh HY, Kim MA, Han MY, Yon DK (2021). Incidence of cancer after asthma development: 2 independent population-based cohort studies. J Allergy Clin Immunol.

[19] Jeon K, Kim SY, Jeong BH, Chang B, Shin SJ, Koh WJ (2013). Severe vitamin D deficiency is associated with non-tuberculous mycobacterial lung disease: a case-control study. Respirology.

[20] Izumi K, Morimoto K, Hasegawa N, Uchimura K, Kawatsu L, Ato M, Mitarai S (2019). Epidemiology of adults and children treated for nontuberculous mycobacterial pulmonary disease in Japan. Ann Am Thorac Soc.

[21] Winthrop KL, Marras TK, Adjemian J, Zhang H, Wang P, Zhang Q (2020). Incidence and prevalence of nontuberculous mycobacterial lung disease in a large US managed care health plan, 2008–2015. Ann Am Thorac Soc.

[22] Cowman S, van Ingen J, Griffith DE, Loebinger MR (2019). Non-tuberculous mycobacterial pulmonary disease. European Respir J.

[23] Lake MA, Ambrose LR, Lipman MC, Lowe DM (2016). ‘” Why me, why now?” Using clinical immunology and epidemiology to explain who gets nontuberculous mycobacterial infection. BMC Med.

[24] Andréjak C, Nielsen R, Thomsen VØ, Duhaut P, Sørensen HT, Thomsen RW (2013). Chronic respiratory disease, inhaled corticosteroids and risk of non-tuberculous mycobacteriosis. Thorax.

[25] Brode SK, Campitelli MA, Kwong JC, Lu H, Marchand-Austin A, Gershon AS, Jamieson FB, Marras TK (2017). The risk of mycobacterial infections associated with inhaled corticosteroid use. European Respir J.

[26] Reich JM, Johnson RE (1992). Mycobacterium avium complex pulmonary disease presenting as an isolated lingular or middle lobe pattern: the lady windermere syndrome. Chest.

[27] Kim RD, Greenberg DE, Ehrmantraut ME, Guide SV, Ding L, Shea Y, Brown MR, Chernick M, Steagall WK, Glasgow CG (2008). Pulmonary nontuberculous mycobacterial disease: prospective study of a distinct preexisting syndrome. Am J Respir Crit Care Med.

[28] Griffith DE, Aksamit T, Brown-Elliott BA, Catanzaro A, Daley C, Gordin F, Holland SM, Horsburgh R, Huitt G, Iademarco MF (2007). An official ATS/IDSA statement: diagnosis, treatment, and prevention of nontuberculous mycobacterial diseases. Am J Respir Crit Care Med.

[29] Ku JH, Ranches G, Siegel SA, Winthrop KL (2020). ‘Lady Windermere's counterpart? Pulmonary nontuberculous mycobacteria in men with bronchiectasis. Diagn Microbiol Infect Dis.

[30] Bhatt SP, Nanda S, Kintzer JS (2009). The lady windermere syndrome. Prim Care Respir J.

[31] Marras TK, Mirsaeidi M, Chou E, Eagle G, Zhang R, Leuchars M, Zhang Q (2018). Health care utilization and expenditures following diagnosis of nontuberculous mycobacterial lung disease in the United States. J Manag Care Spec Pharm.

[32] Lee SW, Kim HC, Nam C, Lee H-Y, Ahn SV, Oh YA, Suh I (2019). Age-differential association between serum uric acid and incident hypertension. Hypertens Res.

[33] Chobanian AV (2007). Isolated systolic hypertension in the elderly. N Engl J Med.

[34] Mori K, Tabusadani M, Yamane K, Takao S, Kuroyama Y, Matsumura Y, Ono K, Kawahara K, Omatsu S, Fujiwara K (2021). Effects of pain on depression, sleep, exercise tolerance, and quality of life in patients with nontuberculous mycobacterial pulmonary disease. Medicine.

[35] Jung HI, Kim S-A, Kim H-J, Yim J-J, Kwak N (2021). Anxiety and depression in patients with nontuberculous mycobacterial pulmonary disease: a prospective cohort study in South Korea. Chest.

[36] Kakuta T, Tabusadani M, Yamane K, Takao S, Kuroyama Y, Mori K, Kawahara K, Ono K, Omatsu S, Senjyu H (2021). Prevalence of depressive symptoms and related risk factors in Japanese patients with pulmonary nontuberculous mycobacteriosis. Psychol Health Med.

[37] Park SC, Kang MJ, Han CH, Lee SM, Kim CJ, Lee JM, Kang YA (2019). Prevalence, incidence, and mortality of nontuberculous mycobacterial infection in Korea: a nationwide population-based study. BMC Pulm Med.

[38] Song JH, Kim BS, Kwak N, Han K, Yim J-J (2021). Impact of body mass index on development of nontuberculous mycobacterial pulmonary disease. European Respir J.

