# Supplementary material for: Comorbidities of nontuberculous mycobacteria infection in Korean adults: results from the National Health Insurance Service–National Sample Cohort (NHIS–NSC) database
Source: BMC Pulm Med. 2022 Jul 23;22:283. doi: 10.1186/s12890-022-02075-y (PMC9308178; doi:10.1186/s12890-022-02075-y)
Supplement: Supplementary file 1 — Additional file 1: Table S1. Comorbidities of nontuberculous mycobacteria infection according to age group (20-39 years old). Table S2. Comorbidities of nontuberculous mycobacteria infection according to age group (40-59 years old). Table S3. Comorbidities of nontuberculous mycobacteria infection according to age group (60-79 years old). Table S4. Comorbidities of nontuberculous mycobacterial infection according to age group (80-89 years old) [file 12890_2022_2075_MOESM1_ESM.zip › Additional File 1.docx]

Supplementary Table 1. Comorbidities of nontuberculous mycobacteria infection according to age group (20-39 years old)

| Comorbidities | NTM (N=188) | |  | Non-NTM (N=752) | | Odds ratio [95% CI]* | p value |
| --- | --- | --- | --- | --- | --- | --- | --- |
|  | n | (%) |  | n | (%) |  |  |
| Diseases of the circulatory system |  |  |  |  |  |  |  |
| Hypertension | 13 | (10.2) |  | 16 | (3.1) | 3.58 [1.67-7.64] | 0.0010 |
| Chronic heart failure | 0 | (0.0) |  | 4 | (0.8) | <0.001 [<0.001->999.99] | 0.9578 |
| Ischemic heart disease | 2 | (1.6) |  | 4 | (0.8) | 2.05 [0.37-11.33] | 0.4107 |
| Arrhythmia | 6 | (4.7) |  | 9 | (1.8) | 2.93 [1.01-8.50] | 0.0474 |
| Endocrine, nutritional and metabolic diseases |  |  |  |  |  |  |  |
| Diabetes mellitus | 20 | (15.6) |  | 22 | (4.3) | 4.15 [2.19-7.86] | <0.0001 |
| Dyslipidemia | 27 | (21.1) |  | 66 | (12.9) | 1.80 [1.10-2.93] | 0.0187 |
| Diseases of the respiratory system |  |  |  |  |  |  |  |
| Acute sinusitis | 89 | (69.5) |  | 266 | (52.0) | 1.69 [1.21-2.36] | 0.0020 |
| Chronic sinusitis | 53 | (41.4) |  | 139 | (27.1) | 1.76 [1.21-2.56] | 0.0031 |
| COPD | 3 | (2.3) |  | 3 | (0.6) | 4.17 [0.92-21.02] | 0.0837 |
| Diffuse pan-bronchiolitis | 2 | (1.6) |  | 2 | (0.4) | 5.30 [0.68-41.38] | 0.1120 |
| Asthma | 56 | (43.8) |  | 119 | (23.2) | 2.31 [1.59-0.63] | <0.0001 |
| Bronchiectasis | 6 | (4.7) |  | 1 | (0.2) | 27.44 [3.24-232.66] | 0.0023 |
| Interstitial pneumonia | 0 | (0.0) |  | 0 | (0.0) | NA | NA |
| Diseases of the musculoskeletal system |  |  |  |  |  |  |  |
| Rheumatoid arthritis | 2 | (1.6) |  | 11 | (2.1) | 0.73 [0.16-3.37] | 0.6898 |
| Osteoporosis | 4 | (3.1) |  | 1 | (0.2) | 18.54 [2.01-170.65] | 0.0099 |
| Bone fracture | 29 | (22.7) |  | 59 | (11.5) | 2.21 [1.36-3.59] | 0.0013 |
| Diseases of the digestive system |  |  |  |  |  |  |  |
| Chronic viral hepatitis | 2 | (1.6) |  | 1 | (0.2) | 8.30 [0.74-92.85] | 0.0860 |
| GERD | 86 | (67.2) |  | 206 | (40.2) | 2.30 [1.64-3.22] | <0.0001 |
| Diseases of the genitourinary system |  |  |  |  |  |  |  |
| Chronic kidney disease | 2 | (1.6) |  | 5 | (1.0) | 1.66 [0.32-8.70] | 0.5492 |
| Diseases of the skin and subcutaneous tissue |  |  |  |  |  |  |  |
| Atopic dermatitis | 22 | (17.2) |  | 51 | (10.0) | 1.87 [1.09-3.18] | 0.0220 |
| Seborrheic dermatitis | 22 | (17.2) |  | 65 | (12.7) | 1.41 [0.84-2.36] | 0.0056 |
| Contact dermatitis | 108 | (84.4) |  | 365 | (71.3) | 1.46 [1.05-2.03] | 0.0264 |
| Other dermatitis | 37 | (28.9) |  | 106 | (20.7) | 1.50 [0.99-2.27] | 0.0572 |
| Urticaria | 62 | (48.4) |  | 170 | (33.2) | 1.71 [1.20-2.44] | 0.0030 |
| Mental and behavioral disorders | 50 | (39.1) |  | 136 | (26.6) | 1.67 [1.14-2.44] | 0.0085 |
| Neoplasms | 6 | (4.7) |  | 5 | (1.0) | 5.49 [1.63-18.54] | 0.0061 |

Abbreviations: COPD, chronic obstructive pulmonary disease; GERD, gastroesophageal reflux disease; NTM, nontuberculous mycobacteria infection

*adjusted for age, sex, house income, and region
