# Supplementary material for: Comorbidities of nontuberculous mycobacteria infection in Korean adults: results from the National Health Insurance Service–National Sample Cohort (NHIS–NSC) database
Source: BMC Pulm Med. 2022 Jul 23;22:283. doi: 10.1186/s12890-022-02075-y (PMC9308178; doi:10.1186/s12890-022-02075-y)
Supplement: Supplementary file 1 — Additional file 1: Table S1. Comorbidities of nontuberculous mycobacteria infection according to age group (20-39 years old). Table S2. Comorbidities of nontuberculous mycobacteria infection according to age group (40-59 years old). Table S3. Comorbidities of nontuberculous mycobacteria infection according to age group (60-79 years old). Table S4. Comorbidities of nontuberculous mycobacterial infection according to age group (80-89 years old) [file 12890_2022_2075_MOESM1_ESM.zip › Additional File 2.docx]

Supplementary Table 2. Comorbidities of nontuberculous mycobacteria infection according to age group (40-59 years old)

| Comorbidities | NTM (N=128) | |  | Non-NTM (N=512) | | Odds ratio [95% CI]* | p value |
| --- | --- | --- | --- | --- | --- | --- | --- |
|  | n | (%) |  | n | (%) |  |  |
| Diseases of the circulatory system |  |  |  |  |  |  |  |
| Hypertension | 72 | (52.5) |  | 240 | (46.9) | 1.23 [0.90-1.67] | 0.1943 |
| Chronic heart failure | 12 | (15.0) |  | 39 | (7.6) | 1.18 [0.60-2.29] | 0.6336 |
| Ischemic heart disease | 32 | (27.9) |  | 69 | (13.5) | 1.90 [1.22-2.97] | 0.0045 |
| Arrhythmia | 21 | (14.9) |  | 41 | (8.0) | 2.10 [1.21-3.62] | 0.0079 |
| Endocrine, nutritional and metabolic diseases |  |  |  |  |  |  |  |
| Diabetes mellitus | 73 | (45.5) |  | 164 | (32.0) | 2.02 [1.47-2.76] | <0.0001 |
| Dyslipidemia | 130 | (62.2) |  | 373 | (72.9) | 1.68 [1.29-2.19] | 0.0001 |
| Diseases of the respiratory system |  |  |  |  |  |  |  |
| Acute sinusitis | 127 | (36.8) |  | 324 | (63.3) | 2.08 [1.59-2.72] | <0.0001 |
| Chronic sinusitis | 103 | (32.2) |  | 214 | (41.8) | 2.45 [1.85-3.26] | <0.0001 |
| COPD | 31 | (26.7) |  | 7 | (1.4) | 19.27 [8.35-44.49] | <0.0001 |
| Diffuse pan-bronchiolitis | 12 | (7.6) |  | 2 | (0.4) | 23.88 [5.29-107.70] | <0.0001 |
| Asthma | 138 | (57.6) |  | 278 | (54.3) | 2.86 [2.19-3.74] | <0.0001 |
| Bronchiectasis | 91 | (36.6) |  | 6 | (1.2) | 99.63 [42.55-233.30] | <0.0001 |
| Interstitial pneumonia | 6 | (5.1) |  | 2 | (0.4) | 12.46 [2.49-62.30] | 0.0021 |
| Diseases of the musculoskeletal system |  |  |  |  |  |  |  |
| Rheumatoid arthritis | 29 | (12.7) |  | 56 | (10.9) | 2.18 [1.35-3.50] | 0.0013 |
| Osteoporosis | 53 | (40.1) |  | 125 | (24.4) | 2.04 [1.37-3.03] | 0.0004 |
| Bone fracture | 59 | (29.5) |  | 149 | (29.1) | 1.71 [1.22-2.39] | 0.0018 |
| Diseases of the digestive system |  |  |  |  |  |  |  |
| Chronic viral hepatitis | 5 | (3.3) |  | 9 | (1.8) | 2.13 [0.70-6.42] | 0.1812 |
| GERD | 179 | (69.0) |  | 469 | (91.6) | 2.37 [1.81-3.09] | <0.0001 |
| Diseases of the genitourinary system |  |  |  |  |  |  |  |
| Chronic kidney disease | 9 | (4.5) |  | 11 | (2.1) | 3.31 [1.35-8.09] | 0.0089 |
| Diseases of the skin and subcutaneous tissue |  |  |  |  |  |  |  |
| Atopic dermatitis | 28 | (11.9) |  | 46 | (9.0) | 2.57 [1.57-4.19] | 0.0002 |
| Seborrheic dermatitis | 36 | (14.5) |  | 98 | (19.1) | 1.53 [1.02-2.29] | 0.0423 |
| Contact dermatitis | 181 | (65.0) |  | 585 | (114.3) | 1.62 [1.25-2.12] | 0.0004 |
| Other dermatitis | 59 | (22.3) |  | 159 | (31.1) | 1.60 [1.15-2.24] | 0.0055 |
| Urticaria | 97 | (35.8) |  | 299 | (58.4) | 1.46 [1.10-1.93] | 0.0081 |
| Mental and behavioral disorders | 136 | (62.7) |  | 434 | (84.8) | 1.49 [1.14-1.94] | 0.0036 |
| Neoplasms | 44 | (29.2) |  | 78 | (15.2) | 2.45 [1.64-3.65] | <0.0001 |

Abbreviations: COPD, chronic obstructive pulmonary disease; GERD, gastroesophageal reflux disease; NTM, nontuberculous mycobacteria infection

*adjusted for age, sex, house income, and region
