# Supplementary material for: Comorbidities of nontuberculous mycobacteria infection in Korean adults: results from the National Health Insurance Service–National Sample Cohort (NHIS–NSC) database
Source: BMC Pulm Med. 2022 Jul 23;22:283. doi: 10.1186/s12890-022-02075-y (PMC9308178; doi:10.1186/s12890-022-02075-y)
Supplement: Supplementary file 1 — Additional file 1: Table S1. Comorbidities of nontuberculous mycobacteria infection according to age group (20-39 years old). Table S2. Comorbidities of nontuberculous mycobacteria infection according to age group (40-59 years old). Table S3. Comorbidities of nontuberculous mycobacteria infection according to age group (60-79 years old). Table S4. Comorbidities of nontuberculous mycobacterial infection according to age group (80-89 years old) [file 12890_2022_2075_MOESM1_ESM.zip › Additional File 3.docx]

Supplementary Table 3. Comorbidities of nontuberculous mycobacteria infection according to age group (60-79 years old)

| Comorbidities | NTM (N=366) | |  | Non-NTM (N=1,464) | | Odds ratio [95% CI]* | p value |
| --- | --- | --- | --- | --- | --- | --- | --- |
|  | n | (%) |  | n | (%) |  |  |
| Diseases of the circulatory system |  |  |  |  |  |  |  |
| Hypertension | 222 | (60.7) |  | 903 | (61.7) | 0.95 [0.75-1.21] | 0.6979 |
| Chronic heart failure | 66 | (18.0) |  | 275 | (18.8) | 0.95 [0.71-1.29] | 0.7534 |
| Ischemic heart disease | 116 | (31.7) |  | 394 | (26.9) | 1.26 [0.98-1.62] | 0.0663 |
| Arrhythmia | 59 | (16.1) |  | 148 | (10.1) | 1.72 [1.24-2.38] | 0.0012 |
| Endocrine, nutritional and metabolic diseases |  |  |  |  |  |  |  |
| Diabetes mellitus | 190 | (51.9) |  | 662 | (45.2) | 1.32 [1.04-1.66] | 0.0206 |
| Dyslipidemia | 243 | (66.4) |  | 852 | (58.2) | 1.42 [1.12-1.81] | 0.0043 |
| Diseases of the respiratory system |  |  |  |  |  |  |  |
| Acute sinusitis | 128 | (35.0) |  | 402 | (27.5) | 1.42 [1.12-1.82] | 0.0046 |
| Chronic sinusitis | 120 | (32.8) |  | 240 | (16.4) | 2.50 1.93-3.24] | <0.0001 |
| COPD | 115 | (31.4) |  | 110 | (7.5) | 6.11 [4.51-8.28] | <0.0001 |
| Diffuse pan-bronchiolitis | 31 | (8.5) |  | 22 | (1.5) | 6.38 [3.62-11.25] | <0.0001 |
| Asthma | 224 | (61.2) |  | 517 | (35.3) | 2.96 [2.33-3.76] | <0.0001 |
| Bronchiectasis | 137 | (37.4) |  | 45 | (3.1) | 20.02 [13.82-29.01] | <0.0001 |
| Interstitial pneumonia | 20 | (5.5) |  | 5 | (0.3) | 17.72 [6.56-47.83] | <0.0001 |
| Diseases of the musculoskeletal system |  |  |  |  |  |  |  |
| Rheumatoid arthritis | 46 | (12.6) |  | 152 | (10.4) | 1.25 [0.88-1.78] | 0.2228 |
| Osteoporosis | 164 | (44.8) |  | 529 | (36.1) | 1.70 [1.28-2.25] | 0.0002 |
| Bone fracture | 112 | (30.6) |  | 391 | (26.7) | 1.22 [0.95-1.57] | 0.1274 |
| Diseases of the digestive system |  |  |  |  |  |  |  |
| Chronic viral hepatitis | 14 | (3.8) |  | 34 | (2.3) | 1.69 [0.89-3.19] | 0.1094 |
| GERD | 264 | (72.1) |  | 759 | (51.8) | 2.42 [1.88-3.11] | <0.0001 |
| Diseases of the genitourinary system |  |  |  |  |  |  |  |
| Chronic kidney disease | 19 | (5.2) |  | 56 | (3.8) | 1.38 [0.81-2.36] | 0.2370 |
| Diseases of the skin and subcutaneous tissue |  |  |  |  |  |  |  |
| Atopic dermatitis | 44 | (12.0) |  | 106 | (7.2) | 1.76 [1.21-2.55] | 0.0031 |
| Seborrheic dermatitis | 56 | (15.3) |  | 170 | (11.6) | 1.38 [1.00-1.93] | 0.0539 |
| Contact dermatitis | 241 | (65.8) |  | 847 | (57.9) | 1.41 [1.11-1.79] | 0.0053 |
| Other dermatitis | 86 | (23.5) |  | 289 | (19.7) | 1.25 [0.95-1.65] | 0.1102 |
| Urticaria | 138 | (37.7) |  | 503 | (34.4) | 1.16 [0.91-1.47] | 0.2283 |
| Mental and behavioral disorders | 243 | (66.4) |  | 850 | (58.1) | 1.45 [1.13-1.86] | 0.0031 |
| Neoplasms | 118 | (32.2) |  | 228 | (15.6) | 2.62 [2.01-3.41] | <0.0001 |

Abbreviations: COPD, chronic obstructive pulmonary disease; GERD, gastroesophageal reflux disease; NTM, nontuberculous mycobacteria infection

*adjusted for age, sex, house income, and region
