# Supplementary material for: Comorbidities of nontuberculous mycobacteria infection in Korean adults: results from the National Health Insurance Service–National Sample Cohort (NHIS–NSC) database
Source: BMC Pulm Med. 2022 Jul 23;22:283. doi: 10.1186/s12890-022-02075-y (PMC9308178; doi:10.1186/s12890-022-02075-y)
Supplement: Supplementary file 1 — Additional file 1: Table S1. Comorbidities of nontuberculous mycobacteria infection according to age group (20-39 years old). Table S2. Comorbidities of nontuberculous mycobacteria infection according to age group (40-59 years old). Table S3. Comorbidities of nontuberculous mycobacteria infection according to age group (60-79 years old). Table S4. Comorbidities of nontuberculous mycobacterial infection according to age group (80-89 years old) [file 12890_2022_2075_MOESM1_ESM.zip › Additional File 4.docx]

Supplementary Table 4. Comorbidities of nontuberculous mycobacterial infection according to age group (80-89 years old)

| Comorbidities | NTM (N=45) | |  | Non-NTM (N=180) | | Odds ratio [95% CI]* | p value |
| --- | --- | --- | --- | --- | --- | --- | --- |
|  | n | (%) |  | n | (%) |  |  |
| Diseases of the circulatory system |  |  |  |  |  |  |  |
| Hypertension | 34 | (75.6) |  | 138 | (76.7) | 0.97 [0.45-2.09] | 0.9340 |
| Chronic heart failure | 10 | (22.2) |  | 53 | (29.4) | 0.67 [0.31-1.47] | 0.3172 |
| Ischemic heart disease | 24 | (53.3) |  | 66 | (36.7) | 2.05 [1.05-4.02] | 0.0369 |
| Arrhythmia | 13 | (28.9) |  | 30 | (16.7) | 2.14 [1.00-4.60] | 0.0510 |
| Endocrine, nutritional and metabolic diseases |  |  |  |  |  |  |  |
| Diabetes mellitus | 29 | (64.4) |  | 88 | (48.9) | 1.88 [0.95-3.71] | 0.0693 |
| Dyslipidemia | 37 | (82.2) |  | 97 | (53.9) | 4.10 [1.79-9.40] | 0.0009 |
| Diseases of the respiratory system |  |  |  |  |  |  |  |
| Acute sinusitis | 13 | (28.9) |  | 37 | (20.6) | 1.60 [0.76-3.37] | 0.2159 |
| Chronic sinusitis | 9 | (20.0) |  | 22 | (12.2) | 1.97 [0.82-4.71] | 0.1297 |
| COPD | 19 | (42.2) |  | 35 | (19.4) | 3.71 [1.71-8.07] | 0.0009 |
| Diffuse pan-bronchiolitis | 5 | (11.1) |  | 7 | (3.9) | 3.12 [0.91-10.65] | 0.0701 |
| Asthma | 28 | (62.2) |  | 72 | (40.0) | 2.47 [1.25-4.86] | 0.0092 |
| Bronchiectasis | 12 | (26.7) |  | 7 | (3.9) | 9.89 [3.51-27.84] | <0.0001 |
| Interstitial pneumonia | 6 | (13.3) |  | 2 | (1.1) | 17.79 [3.23-97.89] | 0.0009 |
| Diseases of the musculoskeletal system |  |  |  |  |  |  |  |
| Rheumatoid arthritis | 8 | (17.8) |  | 19 | (10.6) | 1.88 [0.75-4.72] | 0.1768 |
| Osteoporosis | 23 | (51.1) |  | 77 | (42.8) | 1.52 [0.75-3.11] | 0.2480 |
| Bone fracture | 24 | (53.3) |  | 57 | (31.7) | 2.73 [1.36-5.49] | 0.0050 |
| Diseases of the digestive system |  |  |  |  |  |  |  |
| Chronic viral hepatitis | 2 | (4.4) |  | 1 | (0.6) | 8.64 [0.74-100.75] | 0.0852 |
| GERD | 30 | (66.7) |  | 87 | (48.3) | 2.14 [1.08-4.28] | 0.0304 |
| Diseases of the genitourinary system |  |  |  |  |  |  |  |
| Chronic kidney disease | 3 | (6.7) |  | 9 | (5.0) | 1.29 [0.33-5.04] | 0.7195 |
| Diseases of the skin and subcutaneous tissue |  |  |  |  |  |  |  |
| Atopic dermatitis | 3 | (6.7) |  | 17 | (9.4) | 0.67 [0.19-2.40] | 0.5383 |
| Seborrheic dermatitis | 9 | (20.0) |  | 39 | (21.7) | 0.96 [0.42-2.19] | 0.9179 |
| Contact dermatitis | 34 | (75.6) |  | 112 | (62.2) | 1.89 [0.90-4.00] | 0.0941 |
| Other dermatitis | 13 | (28.9) |  | 45 | (25.0) | 1.23 [0.59-2.59] | 0.5807 |
| Urticaria | 14 | (31.1) |  | 60 | (33.3) | 0.95 [0.47-1.94] | 0.8868 |
| Mental and behavioral disorders | 35 | (77.8) |  | 123 | (68.3) | 1.64 [0.76-3.57] | 0.2103 |
| Neoplasms | 19 | (42.2) |  | 34 | (18.9) | 3.34 [1.60-6.93] | 0.0013 |

Abbreviations: COPD, chronic obstructive pulmonary disease; GERD, gastroesophageal reflux disease; NTM, nontuberculous mycobacterial infection

*adjusted for age, sex, house income, and region
